# Nitrogen-vacancy center magnetic imaging of Fe_3_O_4_ nanoparticles inside the gastrointestinal tract of *Drosophila melanogaster*†

**DOI:** 10.1039/d3na00684k

**Published:** 2023-12-05

**Authors:** Niklas Mathes, Maria Comas, Regina Bleul, Katrijn Everaert, Tobias Hermle, Frank Wiekhorst, Peter Knittel, Ralph A. Sperling, Xavier Vidal

**Affiliations:** a Fraunhofer Institute of Applied Solid State Physics IAF Freiburg Germany niklas.mathes@iaf.fraunhofer.de; b Renal Division, Department of Medicine, Faculty of Medicine and Medical Center, University of Freiburg Hugstetter Straße 55 79106 Freiburg Germany; c Fraunhofer Institute for Microengineering and Microsystems IMM Carl-Zeiss-Str. 18-20 55129 Mainz Germany; d Physikalisch-Technische Bundesanstalt Abbestraße 2-12 Berlin Germany; e Department of Solid State Sciences, Ghent University Krijgslaan 281/S1 Ghent Belgium; f TECNALIA, Basque Research and Technology Alliance (BRTA) Derio 48160 Spain xavier.vidal@tecnalia.com

## Abstract

Widefield magnetometry based on nitrogen-vacancy centers enables high spatial resolution imaging of magnetic field distributions without a need for spatial scanning. In this work, we show nitrogen-vacancy center magnetic imaging of Fe_3_O_4_ nanoparticles within the gastrointestinal tract of *Drosophila melanogaster* larvae. Vector magnetic field imaging based on optically detected magnetic resonance is carried out on dissected larvae intestine organs containing accumulations of externally loaded magnetic nanoparticles. The distribution of the magnetic nanoparticles within the tissue can be clearly deduced from the magnetic stray field measurements. Spatially resolved magnetic imaging requires the nitrogen-vacancy centers to be very close to the sample making the technique particularly interesting for thin tissue samples. This study is a proof of principle showing the capability of nitrogen-vacancy center magnetometry as a technique to detect magnetic nanoparticle distributions in *Drosophila melanogaster* larvae that can be extended to other biological systems.

## Abstract 2

Widefield magnetometry based on nitrogen-vacancy centers enables high spatial resolution imaging of magnetic field distributions without a need for spatial scanning.

## Graphical abstract

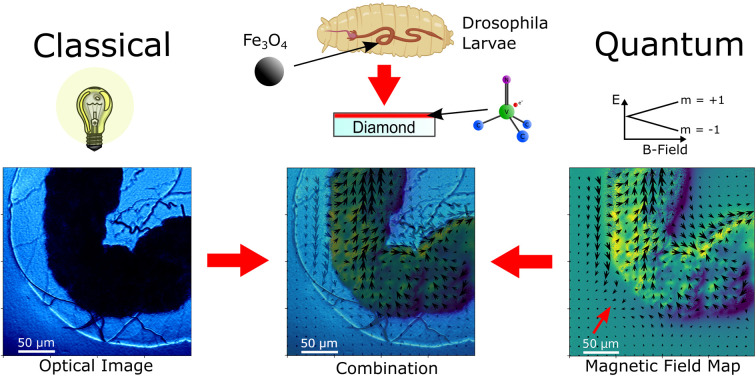

## 1 Introduction

Due to their unique properties, magnetic nanoparticles (MNPs) are of vast interest for experimental biomedical applications.^1–5^ Their extremely small size leads to exceptional magnetic characteristics^6–11^ and enables them to be incorporated into living organisms. Depending on the particle size, a sample of single domain MNPs exhibits a very strong magnetization in the presence of an external magnetic field. Specifically, small MNPs (below approximately 25 nm for ferrimagnetic Fe_3_O_4_ MNPs^10,12^) require very low energy for aligning their magnetic moment. The magnetic moments of very small MNPs fluctuate in the absence of an external field. As a consequence, the collective magnetization of very small MNPs vanishes on larger timescales. Iron oxide nanoparticles are particularly suitable for medical applications^1^ since they are non-toxic^12–14^ and can be excreted by the human organism.^15^ It is possible to functionalize the surface of MNPs in various ways in order to track physiological processes by selectively binding MNPs to relevant molecules (*e.g.* proteins, mRNA). The functionalization enables MNPs to be directed to specific tissues, cells and even organelles within a cell.^16–18^

The increasing importance of MNPs raises a strong interest also in new and improved methods for high spatial resolution magnetic detection.^19^ Magnetic particle imaging (MPI) is a preclinical method for detecting 3D MNP distributions inside a tissue.^20–25^ The technique makes use of the magnetic response generated by the MNPs in a sample placed inside alternating magnetic fields in the kHz range. The measurement signal is collected by sensor coils. Although MPI is very sensitive and offers a good time resolution, the achievable spatial resolution is in the order of a millimeter.^24^ There are efforts to improve the spatial resolution of MPI,^26–28^ but to image the location and distribution of MNPs inside a tissue with higher spatial resolution, a different technique is required that is not limited by sensor coil detection.

Nitrogen-vacancy (NV) centers offer a possible approach to overcome these limitations. NV centers are atomic defects within the diamond crystal lattice consisting of a substitutional nitrogen atom together with an adjacent vacancy. NV center magnetometry is based on the strong response of the color center electronic system to its local environment, in particular, to magnetic fields. The technique offers unique opportunities due to the atomically small size of the color center sensors. NV centers near the surface of a diamond can be placed very close to the target source implying high spatial resolution and a high sensitivity. Since magnetic imaging based on NV centers is a non-toxic and non-destructive technique, it is suitable for biomedical applications.^29–33^ The NV sensor can also be integrated into other equipment, and miniaturization for microfluidic measurements is possible.^34^ Compact and portable NV sensors could be implemented.^35,36^ Although the technique is still under development and not yet established for biomedical applications, there are already a few publications using widefield NV center magnetometry for measuring MNPs,^37–42^ even for MNPs within tissue samples.^43,44^ The technique is not only useful for detecting externally loaded MNPs but also enables measurement of endogenously generated MNPs^33^ or intracellularly stored iron such as in ferritin proteins.^45–47^

In this work we show magnetic imaging of magnetic Fe_3_O_4_ nanoparticles within the gastrointestinal (GI) tract of the *Drosophila melanogaster* larvae using a home-built widefield NV center magnetometer. This technique allows for a diffraction limited spatial resolution of the magnetic imaging based on NV center fluorescence light.^48,49^ This work, which presents the first measurements of MNPs inside full organs known to the authors, is a proof of principle showing that NV center based magnetometry is a powerful method to detect MNPs in biological systems. The motivation to use *Drosophila* as a research model is due to its similarity with the mammalian intestinal epithelia.^50,51^ Although this work does not aim to extract any information about the gastrointestinal function, it shows the potential for further investigation and might help to explore mechanisms as for instance organ–organ communication in mammalian organs, such as the human intestine.

## 2 Experimental methods and techniques

### 2.1 Magnetic nanoparticles

Nanoparticle synthesis of single core magnetic iron oxide nanoparticles was performed by precipitation from alkaline iron chloride solution in an aqueous medium using a continuous micromixer based process as published in ref. 52. Process parameters such as temperature, flow rates and reagents were adjusted to obtain single core MNPs with approximately 29 nm core diameter. MNPs were *in situ* coated with an electrostatic stabilizer and purified by magnetic separation.

**Fig. 1:**
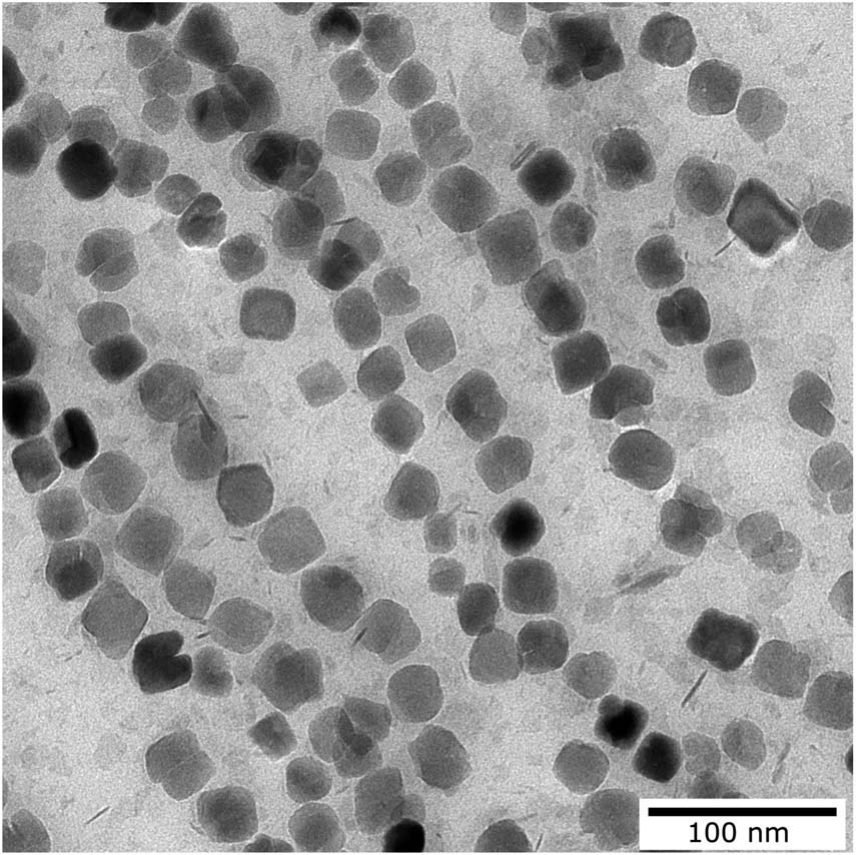
Transmission electron microscopy image of the magnetic nanoparticles used for the measurement.

Core size and shape were determined by transmission electron microscopy (TEM) using a Zeiss Libra 120 electron microscope (Zeiss, Oberkochen, Germany) with an acceleration voltage of 120 kV (Fig. 1). 10 μL of the particle dispersion was loaded onto a carbon coated copper grid and dried at room temperature in the presence of a magnetic field to align the MNPs. Images were recorded using a CCD camera and statistically analyzed using the open-source image processing software ImageJ (National Institutes of Health, Bethesda, MD, USA) to determine the mean diameter, size distribution and standard deviation of particular nanoparticles (*N* > 10^4^ particles). Analytical centrifugation (DCS) was used as an ensemble method to investigate the colloidal stability and hydrodynamic size distribution. DCS measurements were performed at 20 000 rpm (21 504 rcf) (CPS Instruments Inc. Measurements) after calibration with a silicon dioxide standard (255 nm). A sucrose gradient was built using 24% to 8% sucrose. The peak maximum was evaluated using Origin software. The iron content of the MNP samples was determined by the phenanthroline method. After acidic dissolution the formed ferroin complexes were measured photospectroscopically at a wavelength of 510 nm. Similar MNP samples have already been evaluated for other magnetic imaging methods such as magnetic resonance imaging (MRI) and MPI as well as for potential therapeutic intervention by magnet fluid hyperthermia.^53,54^ The iron oxide MNPs used within this work were mainly composed of magnetite (Fe_3_O_4_) and displayed an iron concentration of about 6.5 mM Fe that corresponds to approximately 0.85 μM nanoparticle concentration.

### 2.2 Sample preparation

**Fig. 2:**
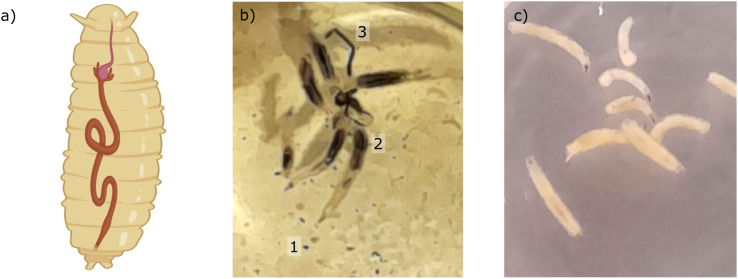
(a) Sketch of the GI tract in a *Drosophila larva* (Image created with https://www.BioRender.com). (b) Picture of *Drosophila larvae* exposed for 24 hours to MNPs. The MNPs show a dark color and can be observed (1) in suspension in the PBS as dots scattered in the well, (2) inside the GI tract of the larva and (3) excreted as feces. (c) Picture of *Drosophila larvae* exposed to the 24 hours PBS control. The average length of *Drosophila melanogaster* 3rd instar larvae is approximately 4 mm.

The *Drosophila melanogaster* flies used for this work were obtained from the Bloomington Drosophila Stock (Bloomington, USA). We applied the UAS/GAL4 system which is the most common method used in *Drosophila* to overexpress or downregulate a gene of interest or in specific tissues.^55^ Specifically, we crossed Dorothy-GAL4 (#6903, BDSC) female flies with UAS-EGFP-RNAi (#41553, BDSC) male flies. We have previously used this cross as an outbred control for transgenic RNAi studies since EGFP-RNAi will not target the expression of an endogenous gene in these flies.^56^ The flies were reared on standard food at 25 °C. We used third-instar larvae, see the sketch of a larva with the GI tract in Fig. 2a. Larvae were placed in 12 well culture plates for 24 hours in phosphate-buffered saline (PBS) containing Fe_3_O_4_ MNPs (100 μg mL^−1^) at 25 °C (see Fig. 2b). The control consisted of the same treatment, but the larvae were placed only in PBS (see Fig. 2c). The larval guts were then dissected out, fixed in 4% paraformaldehyde for 20 minutes and mounted on a microscope coverslip (24 × 50 mm^2^) with a drop of Roti-Mount Carl Roth (Art. No. HP19.1) for *ex vivo* measurements. The diamond plate containing the NV centers was placed on top of the sample with the NV layer side in contact with the larval guts.

### 2.3 Magnetic imaging

The magnetic imaging is based on NV center magnetometry. The NV center in the diamond is a color center within the diamond crystal lattice with already well-known physical properties.^57,58^ The negatively charged NV center (simply called “NV center” in the following text), which possesses an electronic system with a triplet spin ground state, is interesting for quantum sensing because of its unique physical properties. Due to the exceptional characteristics of the diamond host crystal, the NV center electronic quantum states are relatively well defined even at room temperature. The NV center is fluorescent and can be excited using a green laser, the zero phonon line is at 637 nm. After excitation, the NV center electronic system relaxes back to the ground state. At room temperature, transitions in most cases involve the phononic side band. While in most cases the relaxation process is based on fluorescence light emission, there is also an intersystem crossing (ISC) relaxation path. The ISC relaxation does not produce any detectable light and is more likely for the *m*_s_ = ±1 spin states, leading to a reduced fluorescence light emission for the latter states. As a result, the spin quantum state can be detected by measuring the fluorescence intensity. The *m*_s_ = 0 state is called the bright state and the *m*_s_ = ±1 states are called the dark states. A second consequence of the ISC decay path is the possibility to achieve NV center spin initialization to the *m*_s_ = 0 state. The fluorescence transitions are spin conserving but the ISC path alters the spin state ending up almost exclusively in the *m*_s_ = 0. This unique combination of properties enables the NV center quantum system to be used as a nanometer sized sensor.

**Fig. 3:**
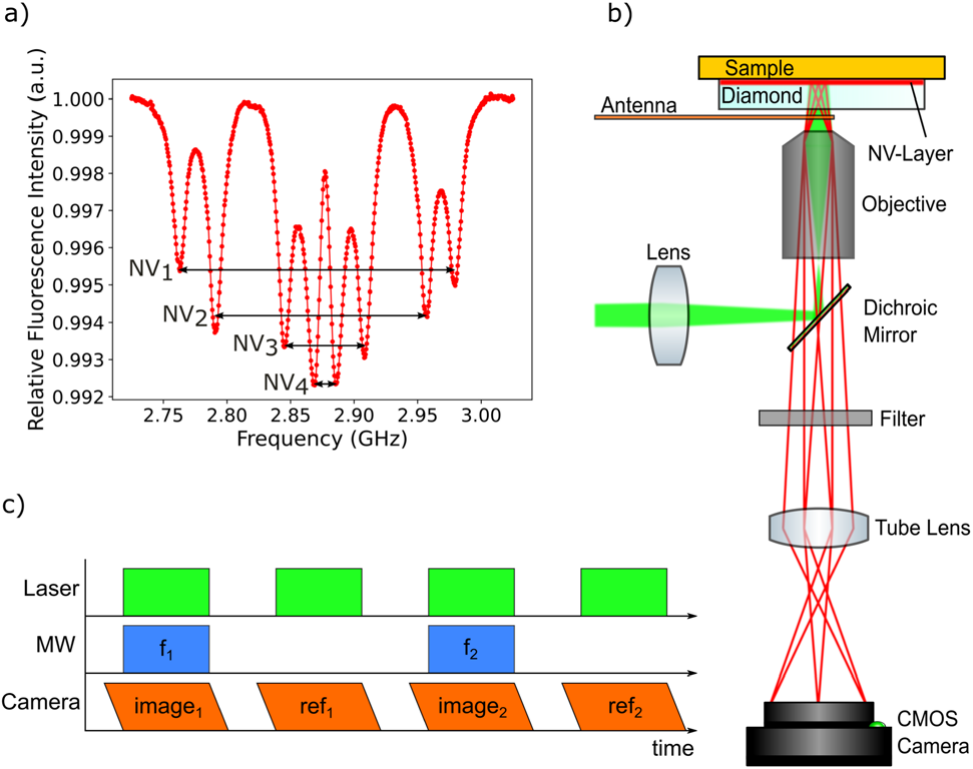
(a) Average ODMR spectrum of all pixels in the field of view. Four pairs of peaks are visible corresponding to the four NV center orientations within the diamond crystal lattice. A bias magnetic field is applied to split all peaks sufficiently for being able to track all eight peak positions. The peaks are broadened due to the change of the magnetic field over the field of view. (b) Sketch of the widefield NV center magnetometry setup. The excitation laser is reflected by a dichroic mirror and focused on the back focal plane of the objective leading to a collimated laser beam illuminating the NV layer. The sample is placed directly on top of the diamond. The fluorescence light emitted from the NV centers is collected by the objective and imaged on a sCMOS camera sensor. Sweeping the frequency of the microwaves supplied by a loop antenna allows an ODMR spectrum to be recorded for all sensor pixels simultaneously. Background light is eliminated using several filters in front of the camera sensor. (c) The cw-ODMR measurement protocol is build in a way to minimize effects from the camera rolling shutter, which is indicated by the angled sides of the camera frames. For each image, the laser and microwaves are only activated when all pixels are exposed. For each frequency step, a reference image is recorded in order to eliminate low-frequent noise.

The magnetic field sensing is carried out using optically detected magnetic resonance (ODMR).^59^ This technique allows local detection of the magnetic field dependent spin energy levels of the NV center electronic system by measuring the fluorescence light. The corresponding measurement protocol is sketched in Fig. 3b. The microwave radiation required for the ODMR protocol is generated using a small loop antenna which is brought close to the diamond. By sweeping the frequency of the microwave radiation, it is possible to detect a drop in fluorescence intensity as soon as the energy difference between the *m*_s_ = 0 and the *m*_s_ = ±1 spin states matches the microwave frequency. Since the *m*_s_ = ±1 spin states are split by an external magnetic field according to the Zeeman effect, the resonance frequencies depend on the magnetic field amplitude at the position of the NV center.

The simplified Hamiltonian of the NV center in an external magnetic field is:^57^

1 *H* = *DS*_*z*_^2^ + *γB⃑*·*S⃑*.

*B* is the external magnetic field, *D* quantifies the zero-field splitting and *γ* is the gyromagnetic ratio of the NV center. *S⃑* = (*S*_*x*_,*S*_*y*_,*S*_*z*_) is the electronic spin-1 operator. The second term describes the Zeeman interaction of the magnetic field with the electronic spin. For small magnetic fields, due to the NV center electronic state symmetry, only the magnetic field component parallel to the NV axis leads to an effect. The resulting Zeeman splitting is approximately proportional to the projection of the magnetic field vector on the main symmetry axis of the NV center:2
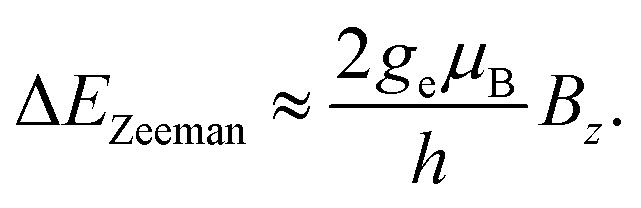
*μ*_B_ is the Bohr magneton, *h* is Planck's constant and *g*_e_ is the *g*-factor of the electron.

While NV center magnetic imaging can be carried out by scanning a single NV center over a surface,^60,61^ we use a near-surface layer of NV centers inside a diamond crystal for sensing. The laser beam is collimated and excites the NV centers within an area of approximately 250 μm in diameter. The fluorescence light emitted by the NV layer is collected by an objective and imaged on a sCMOS camera sensor. This widefield approach allows, compared to scanning NV magnetometry, for a more robust and much faster detection with a larger field of view at the cost of a slightly reduced spatial resolution and a decreased filtering of background light.^48,49^ Widefield NV center magnetic imaging offers a spatial resolution limited by the diffraction of the fluorescence light, which is usually below 1 μm. The field of view is limited by the laser illumination area and can reach the order of millimeters, depending on the imaging magnification.

The NV layer was created by chemical vapor deposition (CVD) overgrowth of a very pure single-crystal diamond.^62,63^ During CVD growth, nitrogen atoms were incorporated into the diamond lattice. The diamond was electron irradiated in order to create vacancies. Afterwards, the diamond was annealed leading to the formation of a dense NV layer. The resulting estimated NV concentration within the layer is about 1 ppm. The thickness of the doped layer is approximately 400 nm as calculated from the CVD growth parameters.

The magnetic imaging of the MNPs inside the larvae GI tract is carried out by placing the tissue sample in between the diamond and a microscope glass slide. The tissue is located very close to the NV layer (the maximum standoff is supposed to be in the order of a few microns) enabling high resolution magnetic imaging. Since there are four different possible orientations of NV centers within the diamond crystal lattice, the ODMR spectrum generally shows eight resonances (see Fig. 3a). In order to separately measure all eight resonance frequencies, a magnetic bias field of about 10 mT is applied with an orientation splitting all resonance peaks. A long working distance air objective is used for the measurements allowing a loop antenna to be placed in between the microscope objective and the diamond plate. As a compromise between high spatial resolution and a large field of view, a 20× magnification objective with a numerical aperture of 0.65 is used. A frequency range of 300 MHz from 2.725 GHz to 3.025 GHz is scanned with 500 kHz steps. The frequency sweep is repeated 30 times and the resulting data for each step are averaged in order to increase the signal-to-noise ratio (Fig. 3c).

The magnetic sensitivity is defined as the smallest detectable magnetic field change. In order to be able to compare measurements with different integration times, the sensitivity is usually normalized to unit time. From the measured ODMR spectrum, the normalized shot-noise limited magnetic sensitivity *η* can be estimated:^64,65^3
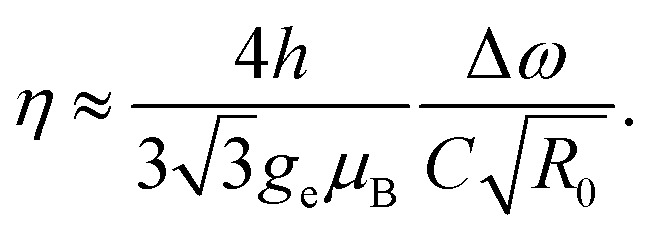
*C* is the ODMR contrast, Δ*ω* is the FWHM resonance linewidth, and *R*_0_ is the base line detection rate.

## 3 Experimental results and discussion

**Fig. 4:**
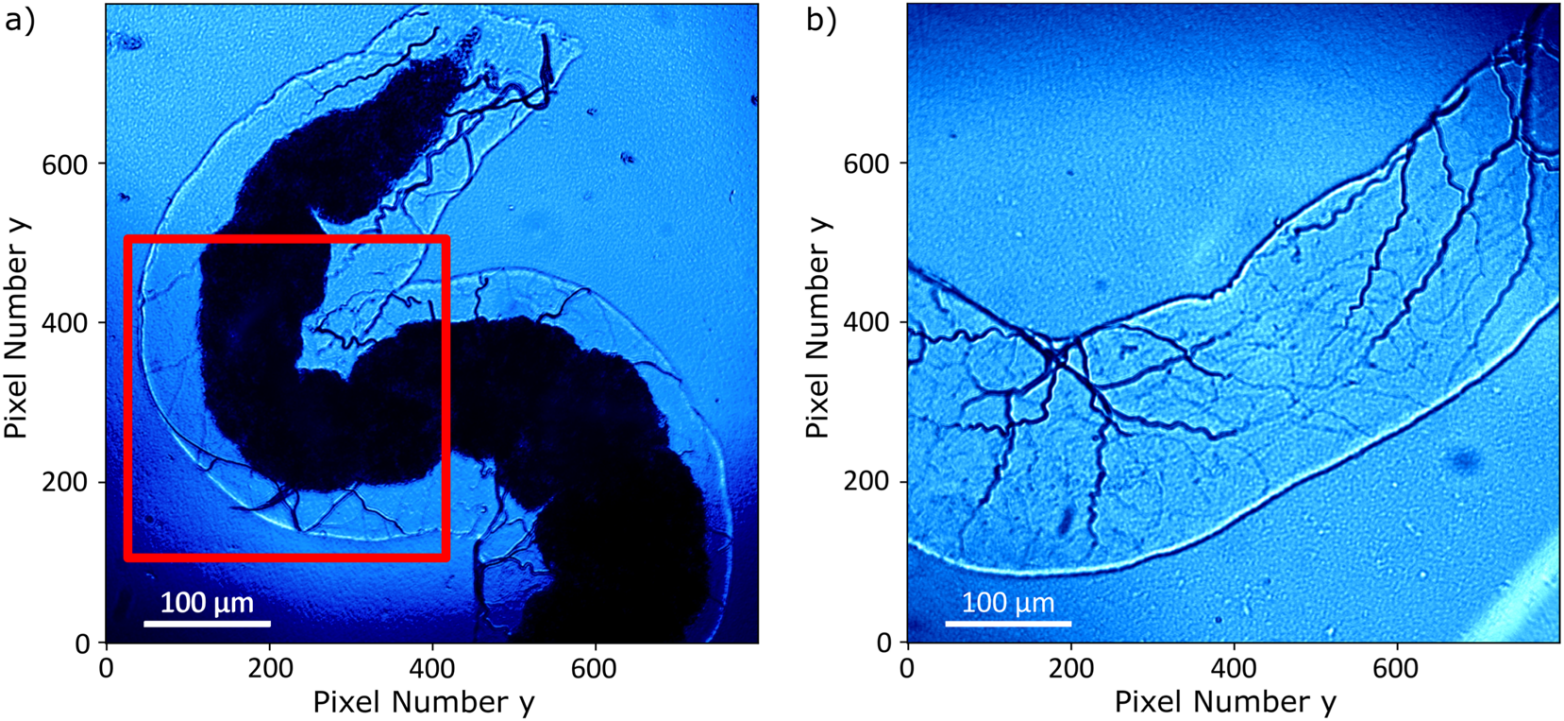
(a) Transmission light image of the measured sample. The intestine organ is clearly visible. The dark area is generated by the accumulated MNPs blocking the light transmission. The area corresponding to the magnetic measurements is indicated by a red square. (b) Transmission light image of a control intestine organ without MNPs.

The *Drosophila melanogaster* larvae were free-fed on Fe_3_O_4_ MNPs for 24 hours with no apparent toxic effects, *i.e.* all larvae survived under experimental conditions as well as in the control until dissection. The magnetic signal generated by Fe_3_O_4_ MNPs accumulated in the larva GI tract (see Fig. 2) is investigated using widefield NV center magnetometry based on ODMR. Before starting the magnetic measurement, a sample area of interest was determined using transmission light microscopy. Different positions on several larvae samples were measured in order to verify reproducibility. The optical transmission image presented in Fig. 4a shows a part of the gut. A 680 nm red LED is used as a backlight. Inside the gut, with a separation of about 40 μm from the outer layer of the organ, a black area is visible. This dark area is generated by the MNPs clustered inside the organ blocking the light transmission. The shape of the black area is similar to the outer shape of the intestine. Other biological tissue structures are visible in the form of thin curly lines and thicker black lines. In Fig. 4b, a transmission light image of an intestine taken from the 24 hours PBS control is presented. As expected, the control image does not show the black area.

**Fig. 5:**
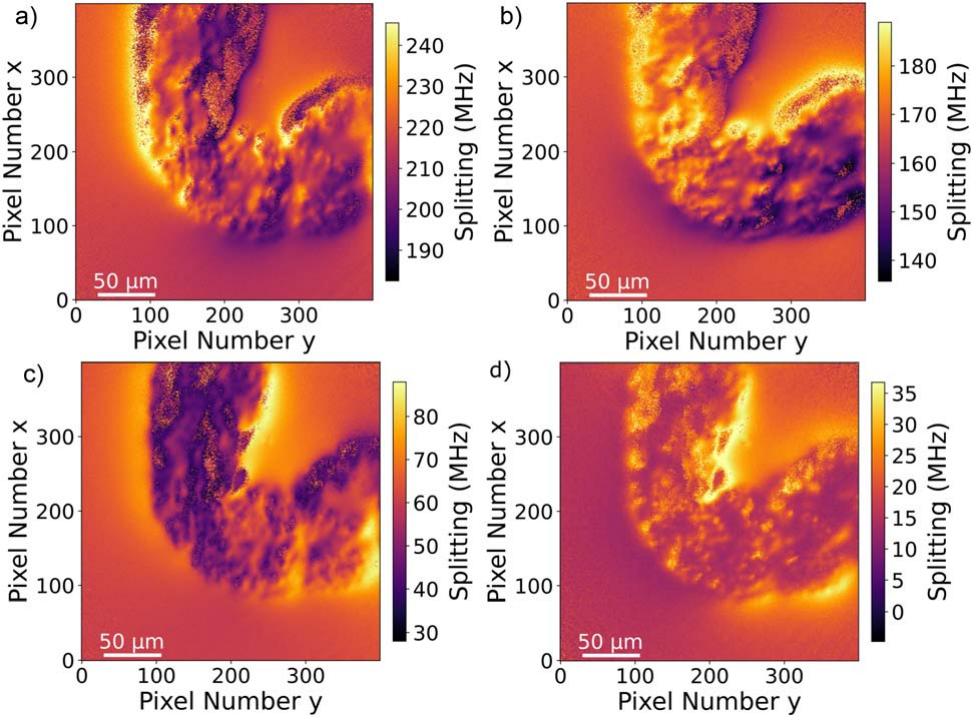
Zeeman splitting maps extracted from the ODMR data for the four NV center orientations within the diamond crystal. Due to the bias magnetic field, the absolute splitting contains corresponding offset values. Due to the strong magnetic field amplitude generated by the MNPs, the peak positions for different orientations slightly overlap in some areas resulting in a noisy image area. Comparing with 3a, (a) corresponds to NV_1_ (b) to NV_2_, (c) to NV_3_ and (d) to NV_4_.

To verify that the black signature inside the intestine is generated by the MNPs we proceed to image the Zeeman splitting of the four NV center orientations as explained above. The resulting Zeeman splitting maps of the four NV center orientations are presented in Fig. 5. The distribution of the MNPs within the intestine organ of the larvae is clearly detectable. The measurement area is approximately (260 × 260) μm^2^. Moreover, there is no other magnetic signal visible in the Zeeman splitting maps. The absence of a magnetic signature from other tissue areas observed within the light microscopy image confirms that the MNPs are contained only inside the intestine. This assessment is given with respect to the magnetic sensitivity of the measurement (see Table 1). The magnetic bias field vector determined from the MNP free area defined by the pixels, 90 to 100 for *x* and *y* respectively, in units of mT is *B⃑*_bias_ = (3.88, 6.08, 4.68). The magnetic field projections onto the NV-center axes show well-defined edges arising from the MNP clusters shaped and curved by the inner tube of the larva gut. Beyond the cluster, the magnetic field does not terminate abruptly but is smoothly directed towards the bias field. Most probably, the signal beyond the edges of the cluster contains an averaged magnetic field generated by the full vertical expansion range of the particle accumulation leading to a smoothing of the field beyond the border. Also the distance of the MNP accumulation is most likely not constant leading to averaging effects of the magnetic signal generated from within a larger area. It can also be observed that the magnetic field orientation differs for the NV center orientations, for instance, in the top central part at the right hand side of the border of the clustered MNPs, NV_1_ and NV_2_ show a magnetic field oppositely oriented with respect to the average value (darker regions indicate a smaller Zeeman Splitting), while the magnetic field projection on NV_3_ and NV_4_ in the same direction shows a wider Zeeman splitting. Globally, the bias magnetic field separates the four magnetic resonances well (see Fig. 3a) and the variation of the magnetic field measured is not strong enough to generate an overlap of any resonances. Nevertheless, in some regions, the resonances are close enough to cause noisy areas in the measurement. Where the resonance peaks are too close, the Lorentzian fitting algorithm fails to recognize the single peaks properly leading to strong deviations of the true value. The differing ODMR contrast for the resonance peak pairs as shown in Fig. 3a is caused by the laser light polarization.

**Table 1:** Lorentzian fitting results together with the calculated shot-noise limited sensitivity and the ODMR signal-to-noise ratio (SNR) for each pair of NV center orientations

	NV_1_	NV_2_	NV_3_	NV_4_
Δ*ω* (MHz)	7.20 ± 1.57	7.93 ± 1.26	7.92 ± 1.37	7.63 ± 1.59
*C* (%)	0.938 ± 0.159	1.165 ± 0.141	1.083 ± 0.141	0.968 ± 0.156
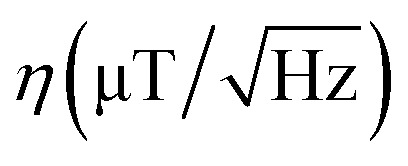	5.78 ± 2.06	5.13 ± 1.32	5.51 ± 1.55	7.65 ± 2.02
SNR (dB)	10.05	11.94	11.30	10.33

For each of the four NV orientations, the theoretical sensitivity is calculated from the peak width and the contrast estimated by Lorentzian fitting of the data captured for each pixel within a small area free of MNPs (pixels 90 to 100 for *x* and *y*, respectively). The fit parameters and the respective deviations are averaged and the sensitivity is calculated using eqn (3). An approximated count rate of 8 million counts per second is assumed by dividing the average count rate of about 40k counts by the exposure time of 5 ms. The results are presented in Table 1. The magnetic sensitivity for the four maps is below 
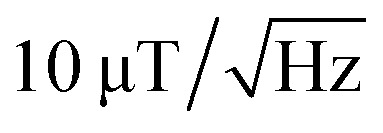
 which is a reasonable value considering the choice of components. The signal-to-noise ratio SNR = 20 log(signal/noise) is calculated for each NV resonance pair by dividing the respective ODMR contrast by the average off-resonance standard deviation estimated for the same pixel area also used for the sensitivity calculations. It would be possible to improve the sensitivity at the cost of setup flexibility and field of view. For example, using a higher numerical aperture (NA) objective would increase the sensitivity because of a more efficient fluorescence light collection. But high NA objectives usually also have a high magnification reducing the measurement field of view. High NA imaging can be achieved using an immersion objective, but those objectives usually have a very small working distance and would therefore only allow working with a very thin diamond plate. Since the MNP magnetic signal is very strong in the presented measurements, the calculated sensitivity can be considered sufficient. The signal-to-noise ratio of the single pixel ODMR measurements is in the order of 10 dB allowing for a sufficiently accurate determination of the peak positions.

**Fig. 6:**
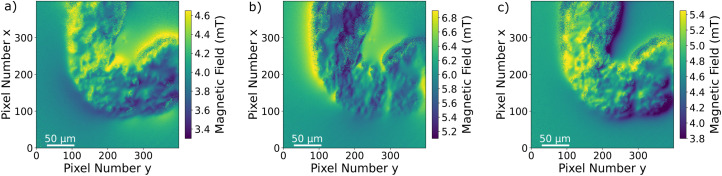
(a) *x*-Component (b) *y*-component and (c) *z*-component of the total magnetic field reconstructed from the Zeeman splitting measured for the four NV center orientations. The shape of the magnetic field distribution is clearly related to the dark area in the light microscopy image. Additionally, modulations of the magnetic field are visible depending on the MNP density and location.

**Fig. 7:**
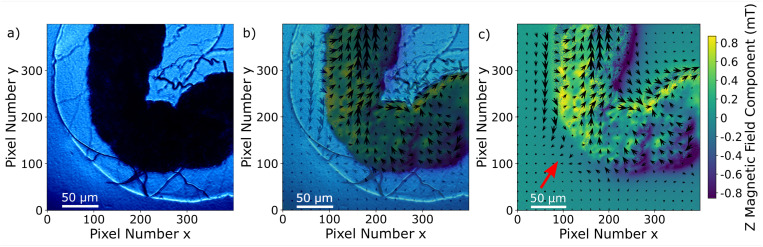
(a) Transmission light microscopy image of the inset marked in a red square in Fig. 4; (b) merging of the transmission light microscopy image with the magnetic field map in (c); (c) magnetic field map, the arrows indicate the *x*–*y* component and the color bar the *z*-component. The bias field was subtracted for all components. The bias field direction is indicated by the red arrow.

Since the Zeeman splitting is proportional to the magnetic field projected onto the respective NV axis and the lattice orientation of the single-crystal diamond is known, it is possible to reconstruct the magnetic field components from the measurement data.^66,67^ This is done by a transformation of the magnetic field vector coordinates. The magnetic field components acquired in the base defined by the four NV center orientations are transformed into the three Cartesian laboratory frame components. Since the orientations of the NV centers within the diamond are known, a transformation matrix can be constructed by defining the Cartesian *z*-axis to be perpendicular to the diamond (100)-surface and the Cartesian *x* and *y*-axes to be parallel to the sides of the diamond plate. The reconstructed magnetic field components resulting from transforming the ODMR maps are presented in Fig. 6a–c. Each magnetic field map contains an offset generated by the bias magnetic field. The magnetic field modulation is at least 1 mT for all components, and for the *y*-component it reaches 1.8 mT. A possible explanation for the stronger modulation in the *y*-direction could be the large bias field component in the same direction. Although the bias field is not very strong, the MNP magnetization is at least partially aligned to the bias field direction. Nevertheless, comparing with the magnetization curves presented in ref. 52, the MNPs should be far from being completely aligned with the external magnetic field as this requires a bias field in the order of 100 mT. The behaviour of the MNPs in the presented measurement is most likely strongly influenced by MNP aggregation and interparticle interaction, and interaction with the tissue can also not be excluded. For instance, the orientation of the outer MNPs of a cluster could be influenced by proximity or contact with the internal layer of the gut. As was already observed in the Zeeman splitting maps, the MNPs accumulated within the organ while there is no magnetic signal from the rest of the sample. In Fig. 7a–c, the transmission microscopy image from the measurement area is faded into a plot representing all three Cartesian magnetic field components. The bias magnetic field offset was subtracted from the respective axes in order to improve the visibility of the MNP magnetic field.

The signal and the accumulation area of the MNPs are well defined as shown in Fig. 7. Although it is not the objective of this manuscript, we realize that a quantification of the MNP density distribution from the measurement is challenging. A quantification would be possible knowing the exact distance of the MNPs to the NV layer or at least the thickness and the density of the accumulated MNPs. Qualitatively, it can be observed that the magnetization of the MNPs predominantly follows the external magnetic bias field but is not fully aligned. This is probably due to the irregular shape and internal structuring of the gut, the local interaction between the wall of the gut and the MNPs, variations in MNP density but mainly due to collective effects of the MNPs. All these contributions should be taken into account for a quantitative evaluation. For example, in the regions around pixels (220, 180) and (380, 260) in Fig. 7c the magnetic field is almost zero due to a magnetic field circulation in the *x*–*y* plane. Similarly, the magnetic field in the upper left quadrant changes sign in the *y*-component from left to right. A deep learning approach might be a solution for a complete understanding of the contribution of each MNP to the total magnetic field.^43^ Measuring the magnetic field generated by MNPs located far from the NV layer results in a less well-defined imaging, not because the imaging itself worsens but due to the magnetic field at the NV layer position being an average of a larger sample area. In other words, increasing the distance between MNPs and the NV layer results in a smoother and therefore less detailed image. A precise slicing of the sample would be a possible solution. Nevertheless, qualitative information on MNP accumulations within tissue samples can already give valuable information on physiological processes.

## 4 Conclusion

The magnetic imaging of MNPs within the *Drosophila melanogaster* larvae shows a very prominent signal within the sample enabling a clear deduction of the MNP accumulation distribution within the GI tract. The measurement allowed full vector information to be acquired with a diffraction limited spatial resolution. In particular, the shape of the detected MNP distribution follows the outer shape of the organ. Clustering effects of the MNPs and the unknown distance of the MNPs to the NV layer do not allow for a quantification of the measurement. Nevertheless, a qualitative knowledge of the accumulation distribution can already be valuable for many applications and pave the way for specialists to draw conclusions about physiological processes. In the future, NV center magnetometry could be used to complement other magnetic field imaging techniques where a high spatial resolution is required or insightful. For example, on a larger scale, the MNP distribution within a tissue sample could be determined using MPI. Sub-areas of interest could be inspected by NV center magnetometry in order to obtain more detailed information about the MNP distribution within the tissue. In combination with MNP surface functionalization,^42^ the magnetic imaging could be used to further explore physiological processes. High spatial resolution imaging of MNPs could also be useful for the further exploration and optimization of various other MNP applications. Currently, MNPs are, for example, used as contrast agents for MRI^68^ or for targeted drug delivery.^3,4,69^ Another promising application is cancer treatment by selective heating of tumor tissue (hyperthermia).^70,71^

To further explore the applicability of NV center magnetometry for MNP detection, a very important step would be to find a way to reliably quantify MNP density distributions. Achieving this goal would signify an important step in the development of applied quantum sensing and would help NV center magnetic imaging to open doors for the visualization and estimation of local interactions influencing the behaviour of MNPs in biological environments. A further step would be the development of a precise measurement protocol allowing for a very accurate reproducibility and repeatability of the measurements. If possible, the NV-based widefield magnetometer development should allow detection of single MNPs.^33^ Ideally, some kind of reference within the sample could be implemented to verify the compliance of the protocol. The measurements presented in this work confirm NV center magnetic imaging as a promising tool for MNP detection in biological systems.

Last but not least, NV-center based quantum sensing is not restricted to DC magnetic field measurements. It is also possible to optically detect alternating magnetic fields at the NV center position using advanced spin manipulation protocols. Since iron oxide magnetic nanoparticles can act as MRI contrast agents, it would be especially interesting to use the NV layer for a high spatial resolution MRI signal detection on a sample similar to the one presented in this work.

## Author contributions

NM and XV conceived the research project and designed the experiments and the instrument. MC and TH designed the protocol for the biological part. RB and RS designed and synthesized the MNPs. PK grew the NV doped diamond. NM carried out the measurements. MC carried out the biological part of the experiment. NM, KE, FW and XV analyzed and validated the data and discussed the results. NM and XV wrote the original draft. MC and RB edited the Experimental methods and techniques section. All authors participated in the discussion and editing of the manuscript.

## Conflicts of interest

There are no conflicts to declare.

## Supplementary Material

NA-006-D3NA00684K-s001
