# Supplementary material for: Nitrogen-vacancy center magnetic imaging of Fe3O4 nanoparticles inside the gastrointestinal tract of Drosophila melanogaster
Source: Nanoscale Adv. 2023 Dec 5;6(1):247–55. doi: 10.1039/d3na00684k (PMC10729879; doi:10.1039/d3na00684k)
Supplement: NA-006-D3NA00684K-s001 [file NA-006-D3NA00684K-s001.pdf]

Electronic Supplementary Material (ESI) for Nanoscale Advances.
This journal is © The Royal Society of Chemistry 2023

Journal Name

ARTICLE TYPE

Cite this: DOI: 00.0000/xxxxxxxxxx

Nitrogen-Vacancy Center Magnetic Imaging of Fe_3O_4 Nanoparticles inside the Gastrointestinal Tract of Drosophila Melanogaster

Niklas Mathes,*[a] Maria Comas,[b], Regina Bleul,[c] Katrijn Everaert,[d,e] Tobias Hermle,[b] Frank Wiekhorst,[d] Peter Knittel,[a] Ralph Sperling,[c] and Xavier Vidal*[a,f]

Electronic Supplementary Information

For the CVD overgrowth, a N/C ratio of 20000 ppm was set resulting in a P1-center concentration of 15 to 17 ppm. The size of the diamond with (100) surface crystal-cut orientation is (4 x 4 x 0.5) mm^3. After the growth, the diamond was electron irradiated at 1 MeV with a dose of $3{\cdot}10^{18}$ cm^{-2} in order to generate vacancies inside the lattice[1]. The diamond was annealed for 2 hours at 950 °C and 10^{-5} mbar. The final NV center concentration is estimated from the approximated conversion rates to be around 1 ppm and the layer thickness is 400 nm. For exciting the NV centers, a 532 nm laser is used. It is pulsed using an acousto-optic modulator, a pinhole is used to remove non-Gaussian modes from the laser beam. The laser beam is focused on the back focal plane of the objective (20X/0.65 NA) resulting in an approximately collimated beam with a Gaussian intensity distribution. Around 70 mW of laser power reach the objective. The $1/e^2$ diameter of the illuminated are is about 300 μm limiting the field of view. The fluorescence light collected by the objective is imaged on a sCMOS camera sensor with a 2 x 2 binned pixel size of (13 x 13) μm^2. The measurement protocol is controlled by an arbitrary wave generator (AWG), which synchronizes camera and AOM driver. The analog output of the AWG generates the microwave signal (sinusoidal, 8 bit, 500 mV_{pp}) fed to the loop antenna while the marker outputs are used for synchronization. The microwave signal is amplified by 45 dB in order to optimize the microwave power with respect to the laser power. For each microwave frequency in the ODMR protocol, one image is recorded together with a microwave free reference image. The first frequency sweep is discarded to remove effects from the thermalization of diamond and sample. The frequency sweep is then repeated 30 times in order to improve the signal-to-noise ratio, resulting in a measurement time of around 10 minutes. The collected data is evaluated on a computer workstation using custom Python code. For each pixel, the spectrum is fit to the sum of eight Lorentzian functions, the peak positions are extracted from the fit parameters. The data evaluation time is reduced by using GPU-accelerated fitting[2].

Notes and references

1 T. Luo, L. Lindner, J. Langer, V. Cimalla, X. Vidal, F. Hahl, C. Schreyvogel, S. Onoda, S. Ishii, T. Ohshima, D. Wang, D. A. Simpson, B. C. Johnson, M. Capelli, R. Blinder and J. Jeske, *New Journal of Physics*, 2022, **24**, 033030.

2 A. Przybylski, B. Thiel, J. Keller-Findeisen, B. Stock and M. Bates, *Scientific Reports*, 2017, **7**, 15722.

[a] *Fraunhofer Institute of Applied Solid State Physics IAF, Freiburg, Germany. E-mail: niklas.mathes@iaf.fraunhofer.de*

[b] *Renal Division, Department of Medicine, Faculty of Medicine and Medical Center, University of Freiburg, Hugstetter Straße 55, 79106 Freiburg, Germany.*

[c] *Fraunhofer Institute for Microengineering and Microsystems IMM, Carl-Zeiss-Str. 18-20, 55129 Mainz, Germany.*

[d] *Physikalisch-Technische Bundesanstalt, Abbestraße 2-12, Berlin, Germany.*

[e] *Department of Solid State Sciences, Ghent University, Krijgslaan 281/S1, Ghent, Belgium.*

[f] *TECNALIA, Basque Research and Technology Alliance (BRTA), 48160, Derio, Spain. E-mail: xavier.vidal@tecnalia.com*
